# Association between multiple chronic conditions and insufficient health literacy: cross-sectional evidence from a population-based sample of older adults living in Switzerland

**DOI:** 10.1186/s12889-023-15136-6

**Published:** 2023-02-06

**Authors:** Maud Wieczorek, Clément Meier, Sarah Vilpert, Robert Reinecke, Carmen Borrat-Besson, Jürgen Maurer, Matthias Kliegel

**Affiliations:** 1 Swiss National Centre of Competence in Research LIVES - Overcoming vulnerability: Life course perspectives, University of Lausanne, Building Géopolis, CH-1015, Lausanne and Geneva, Switzerland; 2 Faculty of Biology and Medicine (FBM), University of Lausanne, Lausanne, Switzerland; 3 Faculty of Business and Economics (HEC), University of Lausanne, Lausanne, Switzerland; 4 Swiss Centre of Expertise in the Social Sciences (FORS), University of Lausanne, Lausanne, Switzerland; 5 Centre for the Interdisciplinary Study of Gerontology and Vulnerability, University of Geneva, Geneva, Switzerland

**Keywords:** Health literacy, Chronic conditions, Multimorbidity, Older adults

## Abstract

**Background:**

Health literacy is the ability to find, understand, assess, and apply health information. Individuals suffering from multiple chronic conditions have complex healthcare needs that may challenge their health literacy skills. This study aimed to investigate the relationship between multimorbidity, the number of chronic conditions, and health literacy levels in a sample of adults aged 58+ in Switzerland.

**Methods:**

We used data from 1,615 respondents to a paper-and-pencil questionnaire administered as part of wave 8 (2019/2020) of the Survey of Health, Ageing and Retirement in Europe (SHARE) in Switzerland. Health literacy was measured using the short version of the European Health Literacy Survey questionnaire. The final score ranged from 0 to 16 and was categorised into three health literacy levels: inadequate (0–8), problematic (9–12), and sufficient (13–16). The number of chronic conditions was self-reported based on a pre-defined list. Associations were examined using multivariable ordinary least squares and ordered probit regression models, controlling for key socio-demographic characteristics.

**Results:**

Overall, 63.5% of respondents reported having at least one chronic condition. Respondents who reported one, two, and three or more chronic conditions were more likely to have lower health literacy scores compared to respondents who did not report any chronic condition (*p<*0.05, *p<*0.01, and *p<*0.001, respectively). Suffering from two and three or more chronic conditions (vs. no chronic condition) was significantly associated with a higher likelihood of having inadequate or problematic health literacy levels (both *p-*values <0.01).

**Conclusions:**

Our findings suggest a need to improve health literacy in older adults suffering from chronic conditions. Improved health literacy could constitute a promising lever to empower individuals to better self-manage their health to ultimately reduce the double burden of chronic diseases and insufficient health literacy in this vulnerable population.

**Supplementary Information:**

The online version contains supplementary material available at 10.1186/s12889-023-15136-6.

## Background

Chronic conditions and multimorbidity cause many challenges in aging populations [1, 2]. At the biological level, aging results from the accumulation of molecular and cellular damages over time [3], which lead to gradual decreases in physical and mental abilities and an increased susceptibility to chronic diseases [4, 5]. In addition to age, chronic diseases have other shared risk factors, including smoking, alcohol consumption, and increased body mass index [6]. Complications of one initial disease can cause other chronic diseases, ultimately leading to multimorbidity [7, 8]. Multimorbidity is associated with subjective cognitive impairment [9], functional decline [10], altered health-related quality of life [11], and an increased risk of mortality [12] in older adults. Besides being a personal burden, chronic conditions represent a complex, long-term challenge for healthcare systems and healthcare providers [13, 14]. The resulting need for novel approaches to manage these conditions more effectively has placed the challenge of good health literacy among the top priorities in public health and policy research [15].

Health literacy is defined as the degree to which individuals have the capacity to find, understand, assess, and apply health information [16]. Recently, the World Health Organization and scientific journal editors called for action to enhance health literacy and identify its correlates, specifically among older adults and other vulnerable groups, including individuals with chronic conditions [17–19]. So far, low health literacy levels have been linked to unfavourable health behaviours and other adverse outcomes, including poorer adherence to physical activity guidelines [20] and medication treatment [21], and an increased number of doctor visits [22] and hospitalizations [23]. Therefore, health literacy constitutes an important determinant of health and healthcare use, especially in the context of chronic conditions and their related complex care needs. Having sufficient health literacy levels is key for individuals to comprehensively manage their chronic conditions daily, to make more autonomous and informed decisions regarding healthcare, and to navigate the healthcare system [24].

Previous cross-sectional studies reported that individuals with chronic conditions or multimorbidity tended to have lower levels of health literacy than individuals with one or no chronic condition [25–29]. Specific difficulties concerned the understanding health information, active engagement with healthcare providers [26] and low levels of critical health literacy (i.e. skills for critically analyzing and reflecting on information or advice received) [29]. This emerging literature currently suffers from three major limitations. First, most of the existing studies included only a limited number of chronic conditions in their assessments. Second, many studies did not specifically focus on older adults, which are specifically vulnerable to (multiple) chronic conditions. Finally, very few studies examined the association between the absolute number of chronic conditions and health literacy levels in older age [26]. The current study set out to address those limitations and aimed to investigate the relationship between multimorbidity, the number of chronic conditions, and health literacy levels in a population-based sample of adults aged 58 years and older living in Switzerland.

## Methods

### Study design and participants

We used data from the Survey of Health, Ageing, and Retirement in Europe (SHARE). SHARE is a multidisciplinary and longitudinal population-based survey of older adults aged 50 and older across 28 European countries and Israel [30]. Individuals who are incarcerated, hospitalized or out of the country during the entire survey period as well as persons who are unable to speak the country’s language(s) or who have moved to an unknown address are excluded from the survey [30]. At each biennial wave, internationally harmonized computer-assisted personal interviewing was used to collect data on health, socioeconomic status, social, family networks, and other life circumstances. In addition, participants were invited to complete a country-specific paper-and-pencil questionnaire.

The present study used data collected during the eighth wave of SHARE Switzerland, from October 2019 to March 2020 [31]. In total, 2,005 older adults living in Switzerland and their partners participated in the face-to-face interviews, and 94% of them (*n=*1,891) also completed a self-administered country-specific questionnaire, which contained an assessment of respondents’ health literacy. At the time of sampling, SHARE Switzerland was designed to be nationally representative of community-dwellers aged 50 and over and their partners. To maintain the representativeness of the sample, periodic refreshments have been performed. Since the last refreshment of the Swiss sample took place in 2011, survey participants aged 50 to 58 in 2019/2020 could only enter SHARE as partners of target respondents such that those survey participants were therefore not representative of the general population aged 50-58. For this reason, the present study only included respondents, or their partners, aged 58 years and over in 2019/2020. After excluding 114 respondents who did not complete the paper-and-pencil questionnaire, 28 respondents younger than 58 years old, and 248 respondents with one or more missing answers on the outcome, exposure variables, or covariates, the final analytical sample consisted of 1,615 individuals.

### Outcomes

The Switzerland-specific paper-and-pencil questionnaire assessed health literacy through the short version of the European Health Literacy Survey questionnaire (HLS-EU-Q16) [32]. This questionnaire consists of 16 items related to concrete health-relevant tasks or situations that respondents rate using a four-point Likert scale ranging from “very easy”, “fairly easy”, “fairly difficult”, to “very difficult”. As described by Pelikan et al., each item was dichotomized, with a value of “0” for the categories “fairly difficult” and “very difficult” and a value of “1” for “very easy” and “fairly easy” [33]. If the overall number of item non-response did not exceed two, missing item values were replaced by 0 [34]. The health literacy total score was calculated by summing the values of each item only for respondents who answered at least 14 items and ranges from 0 to 16. Three categories of health literacy levels were derived from the total score: inadequate health literacy levels (0-8), problematic health literacy levels (9-12), and sufficient health literacy levels (13-16) [33]. Additionally, as health literacy is a multidimensional construct, seven subindices related to three domains (coping with disease/healthcare, disease prevention, health promotion) and four stages of literacy concerning health information processing (accessing health information, understanding health information, processing health information, applying health information) were derived from the scale (Appendix 1). Following an approach suggested by Sørensen et al. [35], the health literacy total score and the seven subindices were standardized to harmonized metrics from 0 to 50 to ease the interpretation and comparison of the results.

### Exposures

The number of chronic diseases and multimorbidity, defined as the coexistence of two or more chronic conditions [36], were the main exposures. Information on diseases and chronic conditions were self-reported. Participants were handed a show card with a list of specific chronic conditions and asked: “Has a doctor ever told you that you had / Do you currently have any of the conditions on this card? With this, we mean that a doctor has told you that you have this condition and that you are either being treated for or bothered by this condition” [31]. The card had 17 conditions and diseases: 1. Heart attack including myocardial infarction or coronary thrombosis or any other heart problem including congestive heart failure, 2. High blood pressure or hypertension, 3. High blood cholesterol, 4. Stroke or cerebral vascular disease, 5. Diabetes or high blood sugar, 6. Chronic lung disease such as chronic bronchitis or emphysema, 7. Cancer or malignant tumour, including leukaemia or lymphoma, but excluding minor skin cancers, 8. Stomach or duodenal ulcer, peptic ulcer, 9. Parkinson’s disease, 10. Cataracts, 11. Hip fracture, 12. Other fractures, 13. Alzheimer’s disease, dementia, organic brain syndrome, senility, or any other serious memory impairment, 14. Other affective or emotional disorders, including anxiety, nervous or psychiatric problems, 15. Rheumatoid Arthritis, 16. Osteoarthritis, or other rheumatism, 17. Chronic kidney disease. The respondents could additionally specify other conditions not mentioned on the list. Any other declared chronic disease was included in the chronic disease count as well.

### Covariates

The key covariates considered in the present study were common socio-demographic variables, including sex (men, women) and age group (58–64 years, 65–74 years, 75+ years). Education levels were grouped into three categories based on the International Standard Classification of Education (ISCED) of 2017 (low, medium, high) [37]. The binary variable for partnership status (has a partner, has no partner) considered all types of partnership. The subjective financial situation of respondents was assessed based on the question: “Is your household able to make ends meet?”. Response categories were recoded as “easily”, “fairly easily” and “with difficulty”. The variable related to respondents’ living area was dichotomized (urban, rural). The language used to answer the questionnaire (German, French, Italian) was used as a proxy for regional/cultural differences.

### Statistical analysis

The cross-sectional weights available in the SHARE dataset were used to calibrate the sample and obtain estimates representative of the target population [31, 38]. The characteristics of the analytical sample were described using unweighted number counts and weighted proportion estimation with corresponding 95% confidence intervals (CI). Consistent with this approach, the distribution of the health literacy levels by exposure variable was examined using weighted proportions and 95% CI. Chi-square tests were used to assess the bivariate associations between the exposure variables and the three categories of health literacy levels. The partial associations between multimorbidity, the number of chronic conditions, and health literacy were examined separately using unweighted ordinary least squares (OLS) regression models (with health literacy total score as outcome) and multivariable ordered probit model (with the three categories of health literacy levels as outcome). The multivariable models thereby accounted for potential confounders including sex, age groups, education levels, partnership status, subjective financial situation, living area, and Swiss linguistic regions. Results were reported as average partial effects along with corresponding standard errors (SE). Additionally, the partial associations between the exposure variables and the standardized health literacy score and its seven subindices were explored using separate unweighted OLS regression models. Since both respondents and their partners could be part of the SHARE study, the possibility of unobserved dependencies between two observations was accounted for in the multivariable models by clustering the estimated standard errors at the household level. Statistical analyses were conducted using STATA/SE 17.0 (STATA Corporation, College Station, TX). Two-sided *p-*values < 0.05 were considered statistically significant.

## Results

### Main characteristics of the analytical sample

Table 1 presents the weighted characteristics of the 1,615 respondents included in the analytical sample. The mean age was 67.5 years (SE: 0.44), half of the respondents were 58-64 years (49.6%), and 51.5% were male. Most of the respondents had a medium education level (63.4%), had a partner (70.9%), lived in the German-speaking part of Switzerland (70.6%), and reported that they could make ends meet easily (56.9%) or fairly easily (30.2%). Their living area was mainly rural (58.3%).

**Table 1:** Main characteristics of the analytical sample, adults aged 58+, SHARE Switzerland, 2019/2020, (*n=*1,615)

		Unweighted	Weighted	
		n	%	95% CI
**Socio-demographic variables**				
Sex	Men	761	51.5	47.3, 55.7
	Women	854	48.5	44.3, 52.7
Age groups	58-64 years	397	49.6	44.6, 54.6
	65-74 years	668	27.2	24.2, 30.5
	75+ years	550	23.1	20.4, 26.0
Education levels	Low	287	15.9	13.1, 19.3
	Medium	1010	63.4	58.8, 67.7
	High	318	20.7	17.0, 24.9
Partnership status	Has a partner	1208	70.9	66.4, 75.0
	No partner	407	29.1	25.0, 33.6
Make ends meet	Easily	892	56.9	52.3, 61.4
	Fairly easily	513	30.2	26.3, 34.5
	With difficulty	210	12.8	10.1, 16.2
Swiss linguistic regions	German	1145	70.6	65.9, 75.0
	French	412	26.4	22.2, 31.2
	Italian	58	3.0	2.0, 4.3
Living area	Urban	739	41.7	37.1, 46.5
	Rural	876	58.3	53.5, 62.9
**Outcomes**				
Health literacy levels	Sufficient	1113	68.8	64.9, 73.1
	Problematic	374	24.6	20.5, 29.1
	Inadequate	128	6.6	4.9, 8.9
**Exposure variables**				
Multimorbidity	No	976	67.2	63.0, 71.2
	Yes	639	32.8	28.8, 37.0
Number of chronic conditions	0	471	36.5	31.6, 41.7
	1	505	30.7	26.6, 35.2
	2	337	17.8	15.0, 20.8
	3	160	7.9	6.1, 10.2
	4	88	3.8	3.0, 4.8
	5	31	1.4	0.9, 2.0
	6	10	0.4	0.2, 0.8
	7	11	0.5	0.2, 0.9
	8	2	1.0	0.2, 4.9

*CI* Confidence intervals

Overall, 93 (5.8%) and 29 (1.8%) respondents had one and two missing HLS-EU-Q16 items, respectively. The respective weighted prevalence of problematic and inadequate health literacy levels was 6.6% and 24.6%. Regarding the exposure variables of interest, almost one third (32.8%) of the respondents suffered from multimorbidity. The count of chronic conditions ranged from 0 (36.5%) to 8 (1.0%). For sample size considerations, respondents who reported suffering from three or more chronic conditions (*n=*302, weighted %: 15.0%) were grouped in one extreme exposure category (3+).

### Weighted distribution of health literacy levels by exposure variable

**Fig. 1:**
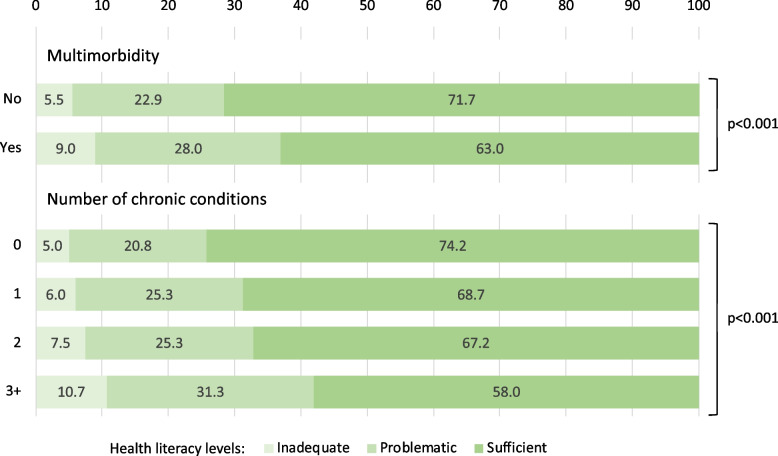
Weighted distribution of health literacy levels in the analytical sample by exposure variable, adults aged 58+, SHARE Switzerland, 2019/2020, *n=*1,615

Figure 1 shows the weighted distribution of health literacy levels, stratified by the two exposure variables. The weighted prevalence of problematic and inadequate health literacy levels was significantly higher among respondents with multimorbidity compared to their counterparts without multimorbidity (*p<*0.001). Similarly, the distribution of the three health literacy levels was significantly different according to the number of reported chronic conditions, with a higher prevalence of problematic and inadequate health literacy levels among respondents with one or multiple chronic conditions (*p<*0.001).

### Multimorbidity, number of chronic conditions, and health literacy

Table 2 describes the partial associations between multimorbidity, the number of chronic conditions, and health literacy from adjusted multivariable models. When controlling for key socio-demographic variables, respondents who suffered from multimorbidity were more likely to have lower health literacy scores than their counterparts without multimorbidity (*p<*0.01). Further, respondents who reported one, two, and three or more chronic conditions were more increasingly likely to have lower health literacy total scores compared to respondents who did not report any chronic disease (*p<*0.05, *p<*0.01, and *p<*0.001, respectively). Similarly, prevalence of multimorbidity and suffering from two and three or more chronic conditions (vs. 0) were associated with a higher likelihood of having inadequate or problematic health literacy levels (all *p-*values <0.01) holding other characteristics fixed.

**Table 2:** Partial associations between multimorbidity, number of chronic conditions and health literacy levels, adults aged 58+, SHARE Switzerland, 2019/2020, *n=*1,615

	Health literacy levels		
	Health literacy score	Problematic vs Sufficient	Inadequate vs Sufficient
**Multimorbidity** (vs no multimorbidity)	-0.48^**^ (0.16)	0.04^**^ (0.01)	0.03^**^ (0.01)
**Number of chronic conditions**			
1 (vs 0)	-0.42^*^	0.04	0.02
	(0.18)	(0.02)	(0.01)
2 (vs 0)	-0.56^**^	0.05^**^	0.03^**^
	(0.21)	(0.02)	(0.01)
3+ (vs 0)	-0.88^***^	0.07^**^	0.04^**^
	(0.25)	(0.02)	(0.01)

The table shows average partial effects and standard errors in parentheses from separate models for multimorbidity and the number of chronic conditions

The ordinary least squares regression and ordered probit regression models control for sex, age partnership status, linguistic region, education levels, subjective financial situation and living area

Concerning the interpretation of the average partial effects, the estimate for multimorbidity in the ordered probit regression, for instance, means that individuals with multimorbidity had a 4-percentage point higher probability of problematic health literacy, compared to individuals without multimorbidity

Statistical significance: * p < 0.05, ** p < 0.01, *** p < 0.001

### Multimorbidity, number of chronic conditions, and health literacy subindices

Average partial associations between multimorbidity, the number of chronic conditions, and health literacy subindices are presented in Table 3. Compared to individuals without multimorbidity, respondents who suffered from multimorbidity were more likely to have lower health literacy scores in the health care and health promotion domains (*p<*0.05 and *p<*0.01, respectively). Also, multimorbidity was associated with lower scores in three of the four stages of literacy concerning information processing, i.e., accessing, understanding, and processing health information (all *p-*values <0.05). Similarly, respondents who reported two and three or more chronic conditions were more likely to have lower health literacy scores concerning most of the three health domains (disease prevention and health promotion) and four stages of literacy concerning health information processing, compared to respondents who did not report any chronic condition.

**Table 3:** Partial associations between multimorbidity, the number of chronic conditions and standardized health literacy subindices, adults aged 58+, SHARE Switzerland, 2019/2020, *n=*1,615

	Health literacy score	Health care	Disease prevention	Health promotion	Access health information	Understand health information	Process health information	Apply health information
**Multimorbidity** (vs no multimorbidity)	-0.98^*^ (0.48)	-0.93^*^ (0.42)	-0.64 (0.47)	-1.63^**^ (0.50)	-1.27^*^ (0.49)	-0.89^*^ (0.40)	-1.25^*^ (0.53)	-0.68 (0.47)
**Number of chronic conditions**								
1 (vs 0)	-0.98^*^	-0.39	-1.08	-1.91^**^	-0.95	-0.72	-1.35^*^	-1.19^*^
	(0.48)	(0.49)	(0.56)	(0.59)	(0.58)	(0.47)	(0.63)	(0.57)
2 (vs 0)	-1.28^*^	-1.06	-0.88	-2.17^**^	-1.40^*^	-1.06^*^	-1.68^*^	-1.16
	(0.54)	(0.54)	(0.64)	(0.68)	(0.63)	(0.54)	(0.72)	(0.65)
3+ (vs 0)	-1.85^**^	-1.23	-1.62^*^	-3.23^***^	-2.23^**^	-1.53^*^	-2.33^**^	-1.50^*^
	(0.60)	(0.64)	(0.68)	(0.73)	(0.74)	(0.61)	(0.78)	(0.70)

The table shows average partial effects and standard errors in parentheses from separate models for multimorbidity and the number of chronic conditions

The ordinary least squares regression models control for sex, age, partnership status, linguistic region, education levels, subjective financial situation and living area

Statistical significance: ^*^ p < 0.05, ^**^ p < 0.01, ^***^ p < 0.001

## Discussion

Using a population-based sample of adults aged 58 and older living in Switzerland, we investigated the relationship between multimorbidity, the number of chronic conditions, and health literacy levels. Multivariable analyses showed that multimorbidity was significantly associated with a higher likelihood of having lower health literacy total scores as well as a higher likelihood of having problematic or inadequate health literacy levels holding other characteristics fixed. Similarly, we observed generally lower levels of health literacy in respondents with more chronic conditions. We further explored associations using the seven health literacy subindices as outcomes, which indicated similar significance and direction of associations overall. Other social, regional, and health determinants of health literacy in the same study population are presented elsewhere [34].

Our findings on the cross-sectional relationship between multimorbidity, the number of chronic conditions and health literacy for older adults in the Swiss general population appear to align with current knowledge, even if inter-study differences in terms of mean age of the study populations, definitions of multimorbidity or chronic conditions, scales used to assess health literacy skills, and the conduct of multivariable analyses somewhat limit direct comparisons across studies. Garcia-Codina et al. reported that having a self-perceived chronic disorder (yes vs. no) was significantly associated with increased odds of having an inadequate or problematic level of health literacy in respondents with a mean age of 46 years [27]. In another study among Finnish adults aged 75 and older, the number of self-reported physician-diagnosed chronic conditions was significantly negatively correlated with health literacy levels [28].

In addition to the use of a more detailed categorization of the number of chronic conditions, one of the original aspects of our study lies in the use of the three health domains and four stages of literacy concerning information processing to get a more holistic picture of individuals' health literacy and its association with (multiple) chronic conditions. So far, few studies have specifically focused on different dimensions of the health literacy construct in the context of multimorbidity and chronic conditions in later life. Heijmans et al. reported that respondents who suffered from multimorbidity were more likely to have lower mean levels of functional health literacy (basic level of reading and writing skills to obtain, understand and use factual information), communicative health literacy (advanced skills that allow a person to extract information, derive meaning from different sources of communication, and apply new information to changing circumstances) and critical health literacy (more advanced skills for critically analyzing and reflecting on information or advice received and using the information to exert greater control over life events and situations), compared to individuals with no or only one chronic condition [29]. In another study, Pedersen et al. found that individuals with more than one physical condition had significantly higher odds of having difficulties specifically in understanding health information and actively engaging with healthcare providers than individuals with only one physical condition [26]. Further, Schaeffer et al. reported that the presence of multiple chronic diseases (more than one health problem persisting for more than six months) was significantly associated with lower health literacy scores only for the “applying health information” stage [25].

We highlight that, independently of key socio-demographic characteristics, older individuals with multiple chronic conditions had lower levels of health literacy in most domains and concerning most information processing stages. Our population-based study, thereby, provides new and more detailed insights on the relationship between health literacy and chronic disease as a major determinant of vulnerability in health. Although our cross-sectional design does not allow us to draw conclusions in terms of causality, our results consistently showed that tasks or situations related to health promotion were particularly challenging for individuals with multimorbidity and for individuals with one, two, and three or more chronic conditions, placing these more vulnerable individuals at particularly high risk for poor disease (self-)management. Conceptually, the health promotion domain includes the ability to stay up*-*to-date concerning one’s health condition, understand health information and derive meaning from it, interpret and evaluate health information, and make informed decisions on health determinants in the social and physical environment [33]. These aspects can have important implications for the target population since major risk factors for the progression of chronic diseases and transition to multimorbidity are generally modifiable. Notably, evidence suggests that lifestyle factors such as smoking, alcohol consumption, physical activity, sedentary lifestyle, and unhealthy diet play an important role in the progression of the most common and deadly chronic diseases [39–42]. Specifically, it has been shown that a healthy lifestyle, including regular physical activity, a healthy diet, no smoking and no or moderate alcohol consumption, was significantly associated with a gain in life years in individuals with multimorbidity [43, 44], suggesting that lifestyle and related prevention behaviours are likely to have a considerable health impact at the individual and population levels. Moreover, previous studies have reported a positive association between health literacy and uptake of at least one health-promoting behaviour in individuals with chronic conditions [45–47], confirming that health literacy is an important factor in the context of disease prevention and control. A growing literature in the developing field of health literacy continues to demonstrate the effectiveness of interventions regarding behaviours that can ultimately decrease disease burden [48, 49]. However, two recently published systematic literature reviews highlighted that the proportion of studies reporting on interventions aimed at improving health literacy in chronic conditions remains low, despite a rapid increase in the number of publications on this health determinant [50, 51]. Since health literacy is a multifaceted and complex issue, a global approach may be necessary to effectively tackle its many challenges. The recent launch of the World Health Organization European Action Network on Health Literacy for Prevention and Control of Noncommunicable Diseases to promote the development of national strategies on health literacy will further support the scaling up of health literacy interventions to improve the implementation of chronic disease prevention and control [52].

While the findings of the present study indicate a clear need to improve health literacy in older adults suffering from one or multiple chronic conditions, several publications called for a wider conceptualization of health literacy, taking into account the complexity of healthcare systems and the availability of understandable health information [15, 19]. An updated definition that acknowledges health literacy as contextual and resulting from several interactions between individuals and the healthcare system has recently been proposed. This definition suggests a conceptual framework where health literacy initiatives are to be multidisciplinary and shared among the diverse stakeholders who influence health behaviours and outcomes [53]. This framework would be particularly relevant for individuals suffering from several chronic conditions since the complexity of their care needs can challenge their health literacy skills. Indeed, health literacy abilities are not always easily transferable to tasks required for effective care of multiple chronic diseases, as understanding co-existent conditions necessitates more advanced health literacy skills, such as in-depth knowledge of pathophysiology or pharmacology. Also, individuals with multiple chronic conditions are sometimes confronted with diverse and potentially conflicting information, multiple treatments, and recommendations from several healthcare providers in diverse settings [54, 55]. Health literacy development should, therefore, be seen as a combined effort between healthcare users and healthcare professionals rather than an individual effort [15, 56]. In this context, measuring health literacy in hospital or primary care settings could be a key lever to personalizing health information and disease management in daily clinical practice. Recent publications developed and validated brief scales to rapidly detect inadequate health literacy in order to better tailor communication to healthcare users’ skills and needs [57–59]. As communication and information exchange constitute a central part of disease management, continued efforts should also be made to support the learning of communication skills throughout the career of health professionals [60]. Additionally, new technologies, such as patient decision support systems linked to electronic health records, may be promising levers for healthcare professionals to provide relevant, individualised health information to healthcare users with multiple chronic conditions [61]. Finally, several systematic reviews and meta-analyses demonstrated the effectiveness of strategies incorporating therapeutic education through different formats such as information handouts, audiovisual offerings, online resources, or building individuals’ skills to encourage shared decision making, improve treatment adherence and mental health outcomes, and empower individuals to better self-manage their health [48, 62].

Although our study contributes valuable insight into the topic, some limitations need consideration. While the study used data from a population-based survey with a high response rate along with population weights to account for differences in selection probabilities and response rates, we cannot exclude the existence of potential residual selection biases. Subpopulations with increased risk of low health literacy, such as the oldest-old adults or individuals with severe health and/or cognitive problems may be underrepresented among the SHARE respondents. In addition, individuals with limited national language skills are not eligible to participate in the Swiss component of the SHARE survey. Furthermore, as the ability to complete a survey can be seen as a health literacy competence, it may be possible that individuals with low health literacy were reluctant to respond to the questionnaire. Therefore, the potential selection biases in this study are likely to have resulted in an overestimation of the health literacy skills of the sample, making the estimates of the observed associations conservative. In addition, the consideration of 17 medically diagnosed diseases in the measurement of chronic conditions does not fully preclude potential bias due to self-reporting. Moreover, although we used a validated and internationally recognized instrument to measure health literacy and its seven related subindices to account for the multidimensional nature of the construct, we cannot rule out that exposure to and need for healthcare and information about health would affect self-reported measures of health literacy. Indeed, individuals who are in good health and have little need for health care may think that they can easily understand oral and written health information, may be unaware of potential difficulties if they actually needed such information and therefore may overestimate their health literacy levels. Also, since we did not adjust for multiple comparison testing, the results when using the standardized health literacy subindices as outcomes should be interpreted with caution. In addition, our study's cross-sectional design did not allow us to assess whether the co-existence of chronic conditions and their association with insufficient health literacy levels change over time; hence, this question remains to be investigated in future studies. Finally, our study exclusively used subjective measures of health literacy. While subjective measures of health literacy like the HLS-EU-Q16 are well-established, commonly used in the literature [63] and likely to be important drivers of health outcomes in part due to their close link with self-efficacy concerning health-related literacy tasks [64], it would nonetheless be interesting to further test the consistency of our findings using performance-based (objective) measures of health literacy such as the Test of Functional Health Literacy in Adults (TOFHLA) [65] or the Newest Vital Sign [66].

## Conclusion

The present study's findings indicate a clear need to improve health literacy in older adults suffering from one or multiple chronic conditions. Improved health literacy could constitute a promising lever to empower individuals to better self-manage their health. Implementing health literacy screening in clinical care could allow for a tailoring and personalization of health information and patient-provider communication, including therapeutic education, with the ultimate goal of reducing the impact of insufficient health literacy on individuals’ health, functional status, and healthcare use in this vulnerable population.

## Supplementary Information

**Additional file 1.**

## Data Availability

The datasets generated and/or analysed during the current study are available to the scientific community upon submitting a data requestion application to the SHARE study (https://share-eric.eu/data/become-a-user). Additional materials can be received upon request on: maud.wieczorek@unil.ch.

## Abbreviations

CI: Confidence Intervals
HLS-EU-Q16: 16-item European Health Literacy Survey Questionnaire
ISCED: International Standard Classification of Education
OLS: Ordinary Least Squares
SE: Standard Errors
SHARE: Survey of Health, Ageing, and Retirement in Europe
