# Supplementary material for: Association between multiple chronic conditions and insufficient health literacy: cross-sectional evidence from a population-based sample of older adults living in Switzerland
Source: BMC Public Health. 2023 Feb 6;23:253. doi: 10.1186/s12889-023-15136-6 (PMC9901105; doi:10.1186/s12889-023-15136-6)
Supplement: Supplementary file 1 — Additional file 1. [file 12889_2023_15136_MOESM1_ESM.docx]

**Supplementary file**

**Association between multiple chronic conditions and insufficient health literacy: cross-sectional evidence from a population-based sample of older adults living in Switzerland**

Maud Wieczorek, Clément Meier, Sarah Vilpert, Robert Reinecke, Carmen Borrat-Besson, Jürgen Maurer, Matthias Kliegel

Appendix 1. Matrix of subindices of health literacy derived from the short version of the European Health Literacy Survey questionnaire (HLS-EU-Q16)

| First, we would like to ask you how comfortable you feel when dealing with health-related information.  For you, how easy or difficult is it to…  Answer categories: "Very easy", "Fairly easy", "Fairly difficult", "Very difficult" |
| --- |

|  | **Access health information** | **Understanding health information** | **Process health information** | **Apply health information** |
| --- | --- | --- | --- | --- |
| **Health care** | Find information on treatments of illnesses that concern you?    Find out where to get professional help when you are ill? | Understand what doctor says to you?  Understand your doctor's or pharmacist's instructions on how to take a prescribed medicine? | Judge when you may need to get a second opinion from another doctor? | Use the information the doctor gives you to make decisions about your illness?  Follow instructions from your doctor or pharmacist? |
| **Disease prevention** | Find information on how to manage mental health problems like stress or depression? | Understand health warnings about behaviour such as smoking, low physical activity, and drinking too much?  Understand why you need health screenings? | Judge if the information on health risks in the media is reliable? | Decide how you can protect yourself from illness based on information in the media? |
| **Health promotion** | Find out about activities that are good for your mental well-being? | Understand advice on health from family members or friends?  Understand information in the media on how to get healthier? | Judge which everyday behaviour is related to your health? |  |
